# Supplementary figures and images for: Widespread gene duplication and adaptive evolution in the RNA interference pathways of the Drosophila obscura group
Source: BMC Evol Biol. 2019 May 8;19:99. doi: 10.1186/s12862-019-1425-0 (PMC6505081; doi:10.1186/s12862-019-1425-0)

# *Drosophila pseudoobscura*

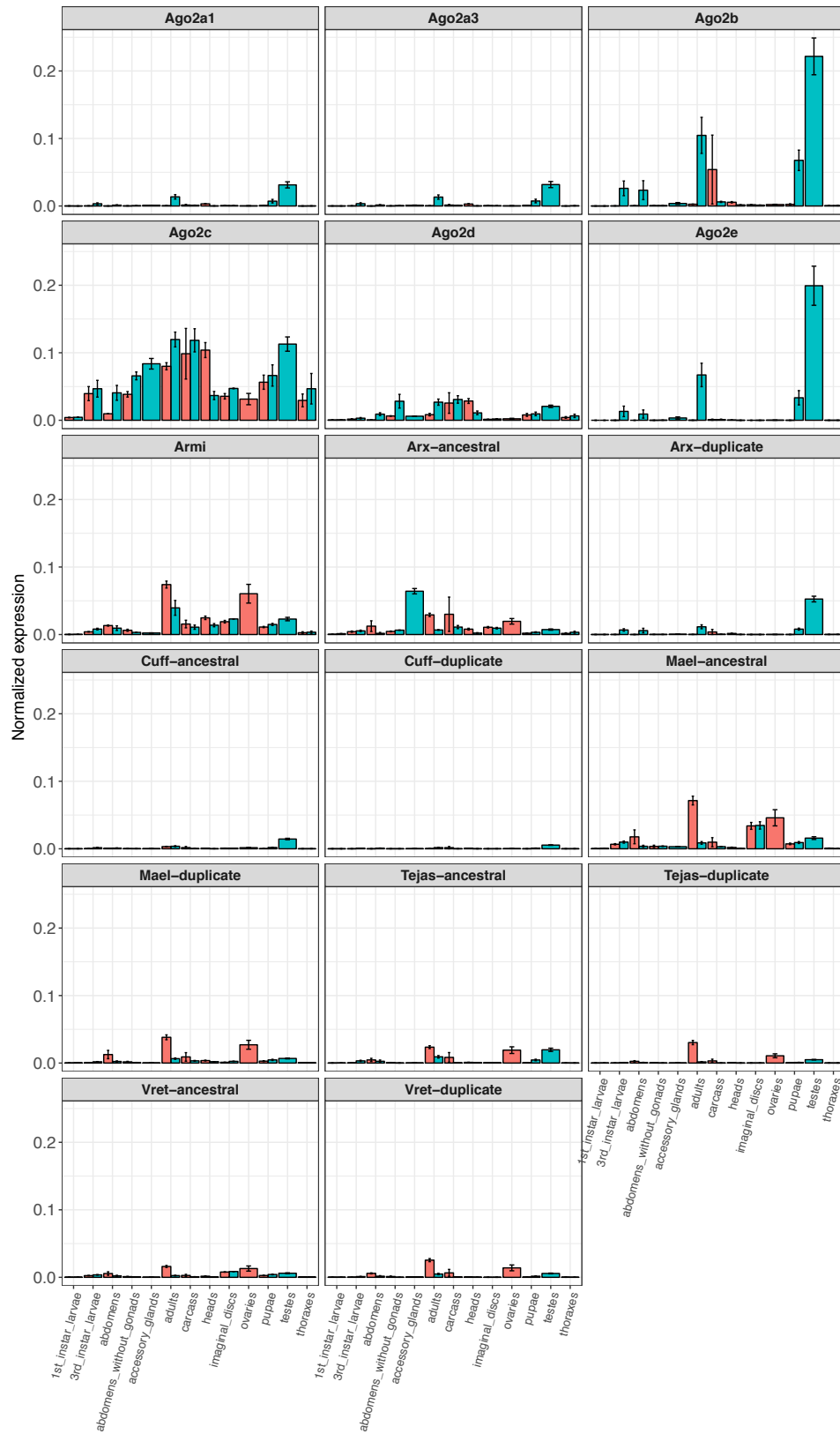

*Drosophila miranda*

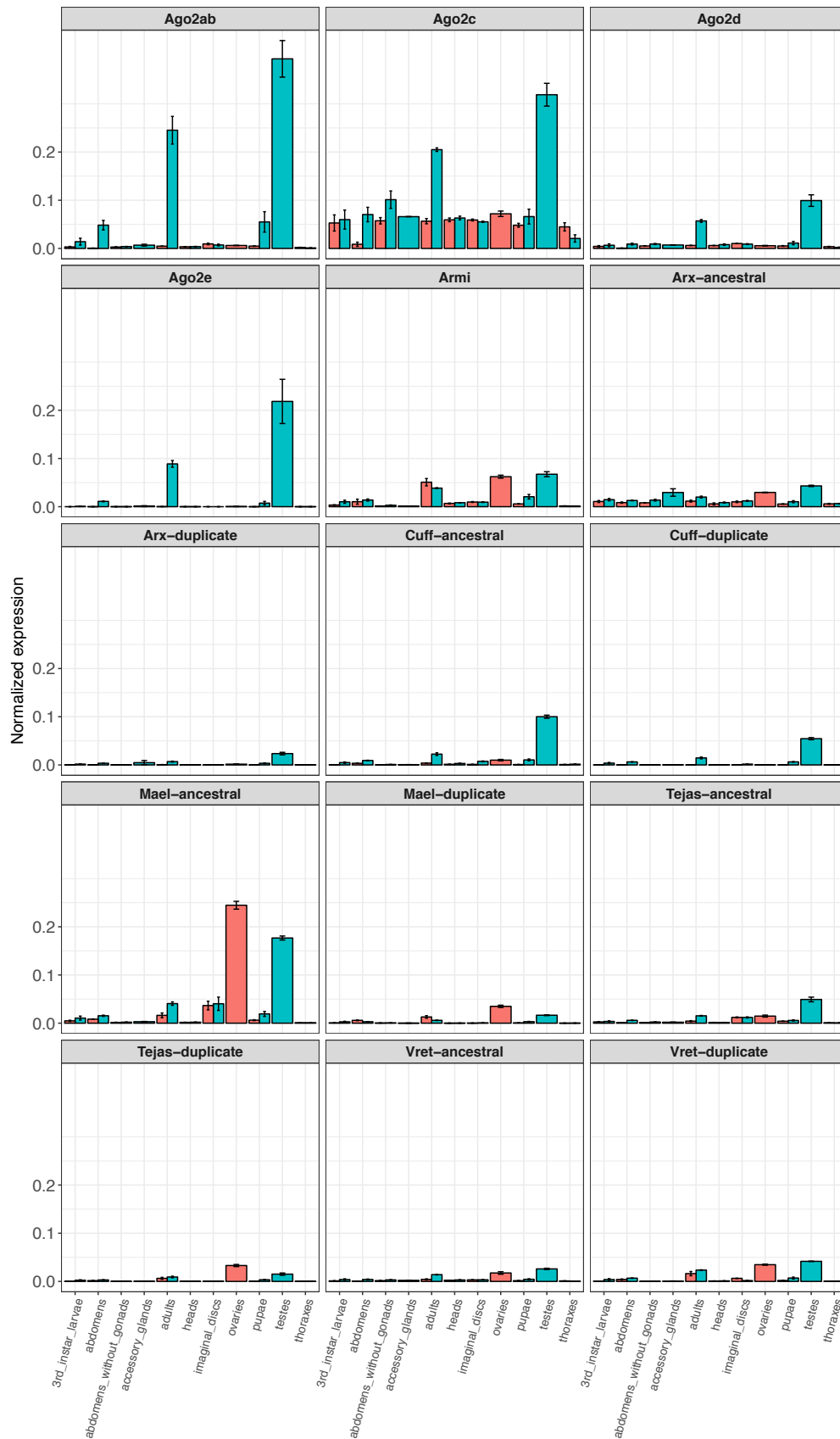

*Drosophila obscura*

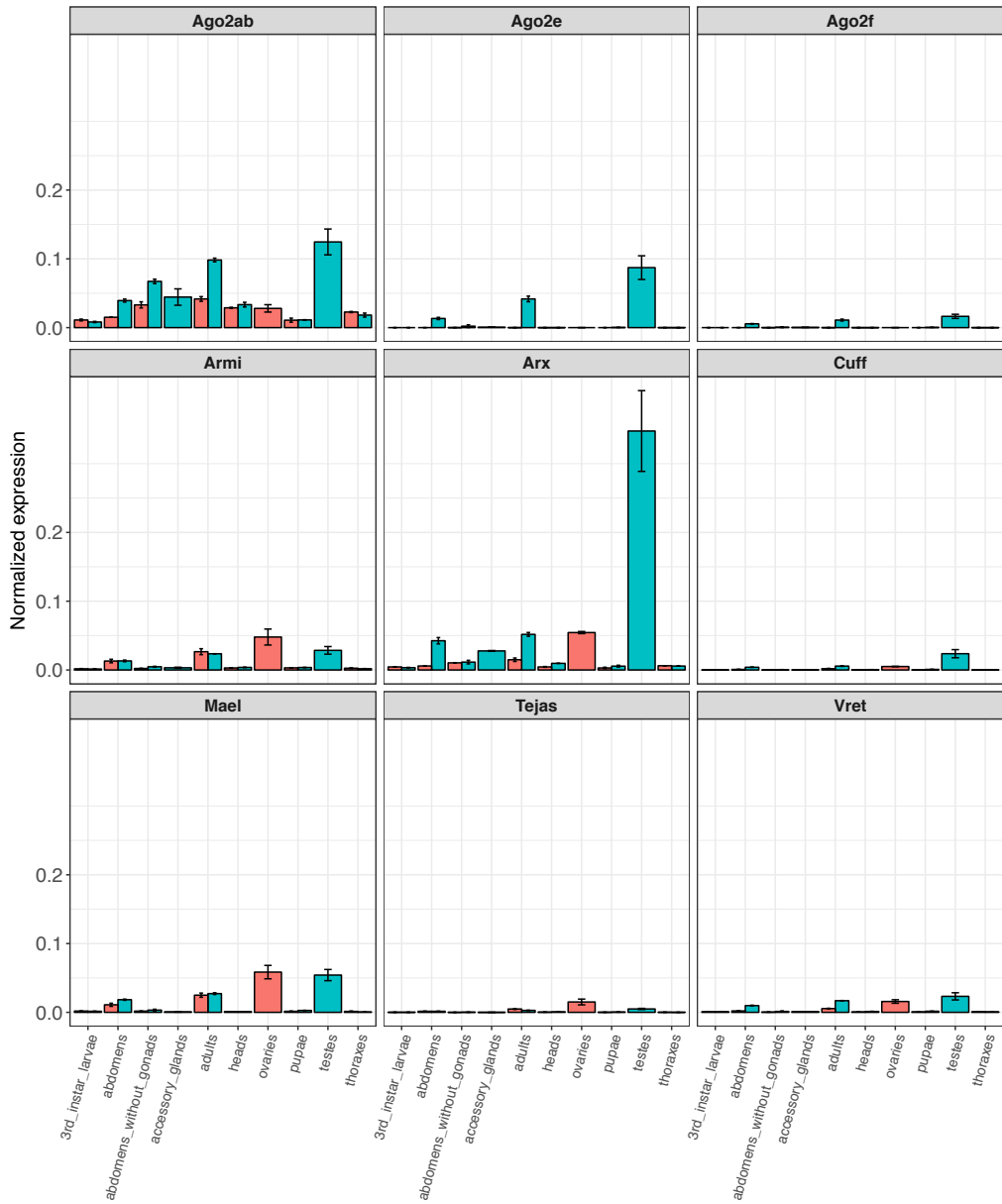

Supplement: Supplementary file 2 — Figure S1. The expression profile of RNAi across tissue. The normalized expression plotted across tissues. The error bars denote the standard error for the given tissue. Blue bar indicates male and female is indicated by red bar. The plot is shown for D. pseudoobscura (n = 163), D. miranda (n = 42) and D. obscura (n = 34), respectively. (PDF 131 kb) [file 12862_2019_1425_MOESM2_ESM.pdf]
