# Supplementary material for: Widespread gene duplication and adaptive evolution in the RNA interference pathways of the Drosophila obscura group
Source: BMC Evol Biol. 2019 May 8;19:99. doi: 10.1186/s12862-019-1425-0 (PMC6505081; doi:10.1186/s12862-019-1425-0)
Supplement: Supplementary file 5 — Figure S2. The heat-map p-value of the difference between pairwise posterior distributions. Large p-value (> 0.05) indicates the overlapping distribution and the duplication time might be shared (blue box). Red boxes denote comparison with p-value < 0.05 which indicate non-overlapping posterior distribution and an asynchronous duplication event. Pink colored box indicates the marginally significant (0.01 < p-value < 0.05). (PDF 221 kb) [file 12862_2019_1425_MOESM5_ESM.pdf]

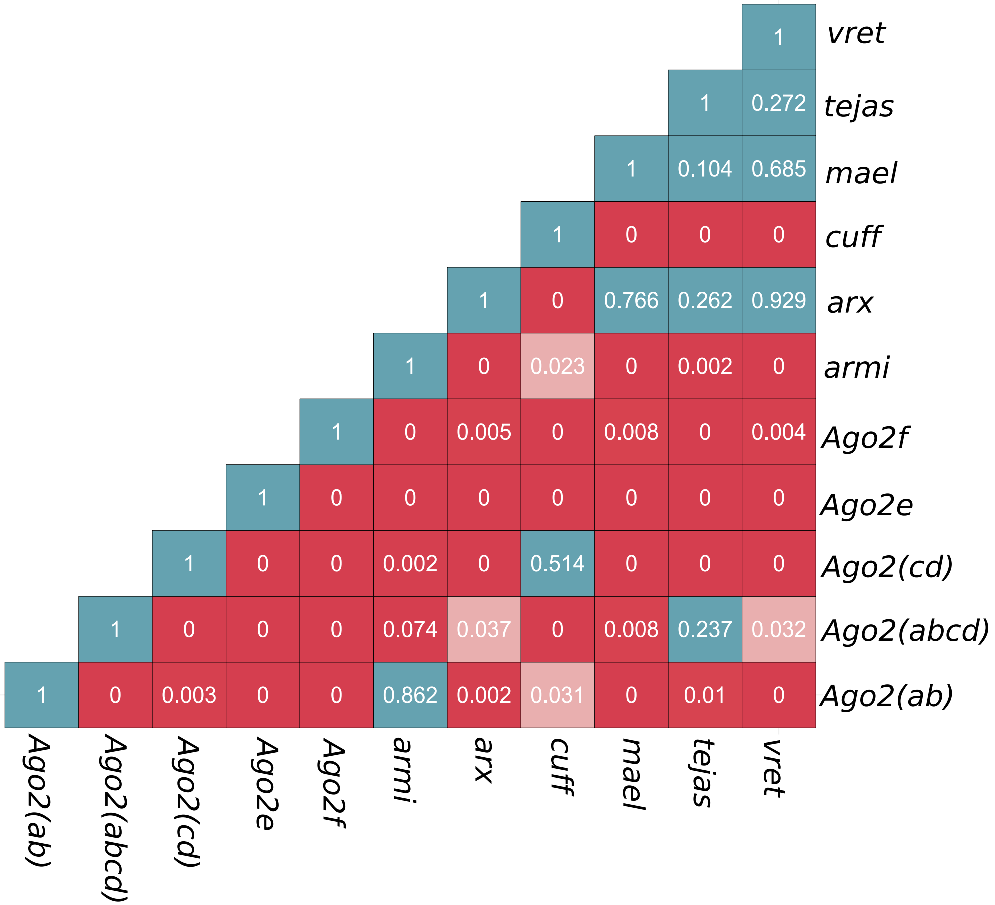
vret 1
tejas 1 0.272
mael 1 0.104 0.685
cuff 1 0 0 0
arx 1 0 0.766 0.262 0.929
armi 1 0 0.023 0 0.002 0
Ago2f 1 0 0.005 0 0.008 0 0.004
Ago2e 1 0 0 0 0 0 0 0
Ago2(cd) 1 0 0 0.002 0 0.514 0 0 0
Ago2(abcd) 1 0 0 0 0.074 0.037 0 0.008 0.237 0.032
Ago2(ab) 1 0 0.003 0 0 0.862 0.002 0.031 0 0.01 0
Ago2(ab)
Ago2(abcd)
Ago2(cd)
Ago2e
Ago2f
armi
arx
cuff
mael
tejas
vret
